# Acute Infections and Environmental Exposure to Organochlorines in Inuit Infants from Nunavik

**DOI:** 10.1289/ehp.7255

**Published:** 2004-08-18

**Authors:** Frédéric Dallaire, Éric Dewailly, Gina Muckle, Carole Vézina, Sandra W. Jacobson, Joseph L. Jacobson, Pierre Ayotte

**Affiliations:** ^1^Department of Social and Preventive Medicine, Laval University, and Public Health Research Unit, CHUQ-Laval University Medical Center, Québec, Canada; ^2^Department of Psychiatry and Behavioral Neurosciences, and; ^3^Department of Psychology, Wayne State University School of Medicine, Detroit, Michigan, USA

**Keywords:** cord blood, environmental health, gastrointestinal infections, human, infant, infections, Inuit, organochlorines, otitis, pesticides, polychlorinated biphenyls, prenatal exposure, respiratory tract infections

## Abstract

The Inuit population of Nunavik (Canada) is exposed to immunotoxic organochlorines (OCs) mainly through the consumption of fish and marine mammal fat. We investigated the effect of perinatal exposure to polychlorinated biphenyls (PCBs) and dichlorodiphenyldichloroethylene (DDE) on the incidence of acute infections in Inuit infants. We reviewed the medical charts of a cohort of 199 Inuit infants during the first 12 months of life and evaluated the incidence rates of upper and lower respiratory tract infections (URTI and LRTIs, respectively), otitis media, and gastrointestinal (GI) infections. Maternal plasma during delivery and infant plasma at 7 months of age were sampled and assayed for PCBs and DDE. Compared to rates for infants in the first quartile of exposure to PCBs (least exposed), adjusted rate ratios for infants in higher quartiles ranged between 1.09 and 1.32 for URTIs, 0.99 and 1.39 for otitis, 1.52 and 1.89 for GI infections, and 1.16 and 1.68 for LRTIs during the first 6 months of follow-up. For all infections combined, the rate ratios ranged from 1.17 to 1.27. The effect size was similar for DDE exposure but was lower for the full 12-month follow-up. Globally, most rate ratios were > 1.0, but few were statistically significant (*p* < 0.05). No association was found when postnatal exposure was considered. These results show a possible association between prenatal exposure to OCs and acute infections early in life in this Inuit population.

Substantial information concerning the contamination of northern marine food by organochlorines (OCs) is now available (Braune et al. 1999; Burkow and Kallenborn 2000; Muir et al. 1999). This family of compounds includes chlorinated pesticides [dichlorodiphenyltrichloroethane (DDT), dieldrin, mirex, and toxaphene] and industrial compounds [hexachlorobenzene (HCB) and polychlorinated biphenyls (PCBs)]. Several OCs are chemically stable. They are thus resistant to biodegradation and can accumulate in adipose tissue of living organisms. This leads to their biomagnification in the aquatic and terrestrial food chain, resulting in the highest levels in top predator species (Braune et al. 1999; Evans et al. 1991; Muir et al. 1999; Skaare et al. 2000). The manufacture of most OCs was halted in the 1970s when regulatory actions were adopted to limit their production and use. Today, OCs are still released into the environment due to improper storage and ongoing use in certain parts of the world.

For cultural and economical reasons, carnivorous fish and marine mammals constitute an important part of the diet of the Inuit population of Nunavik (northern Québec, Canada). Their exposure to bio-magnified substances, such as OCs, is thus proportionally high. Several studies have identified markedly higher concentrations of OCs in adult blood, umbilical cord blood, and breast milk of Nunavik inhabitants compared with those of the southern Québec population (Ayotte et al. 1997, 2003; Dewailly et al. 1989, 1993, 1998; Muckle et al. 1998, 2001; Rhainds et al. 1999).

Exposure to most OCs produces a wide variety of immunotoxic effects in animals and humans. OCs that have a chemical structure similar to 2,3,7,8-tetrachlorodibenzo-*p*-dioxin, such as dioxin congeners and coplanar PCBs, are especially immunotoxic. Alterations of T-cell subsets, of serum IgA and IgM concentrations, of delayed-type hypersensitivity, and of complement function, have been documented in primates and humans (Belles-Isles et al. 2002; Chang et al. 1982; Hoffman et al. 1986; Lu and Wu 1985; Neubert et al. 1992; Tryphonas et al. 1991a, 1991b). The development of the immune system *in utero* and during infancy is particularly sensitive to immunotoxic agents. High exposure during early life could lead to permanent defects in the immune system and thus decrease resistance to infectious agents (Badesha et al. 1995).

The high incidence of acute infectious diseases in infants and children from Nunavik has been known for many years (Bruneau et al. 2001; Dufour 1988; Proulx 1988; Thérien 1988). In this context, we hypothesized that the incidence of infections among Inuit infants was related in part to the high maternal body burden of immunotoxic food-chain contaminants during pregnancy. In 2000, we published a first study on susceptibility to infection in Inuit infants recruited between 1989 and 1990 (Dewailly et al. 2000). We found that the risk of acute otitis media and recurrent otitis media was positively associated with prenatal exposure to OCs. However, postnatal exposure was not considered, and some potential confounding factors could not be evaluated. To confirm the association observed, we investigated the association between exposure to OCs and the incidence rate of acute infections during the first year of life in a second cohort of 199 Inuit infants recruited between 1995 and 2001.

## Material and Methods

### Study population and recruitment.

**Figure 1:**
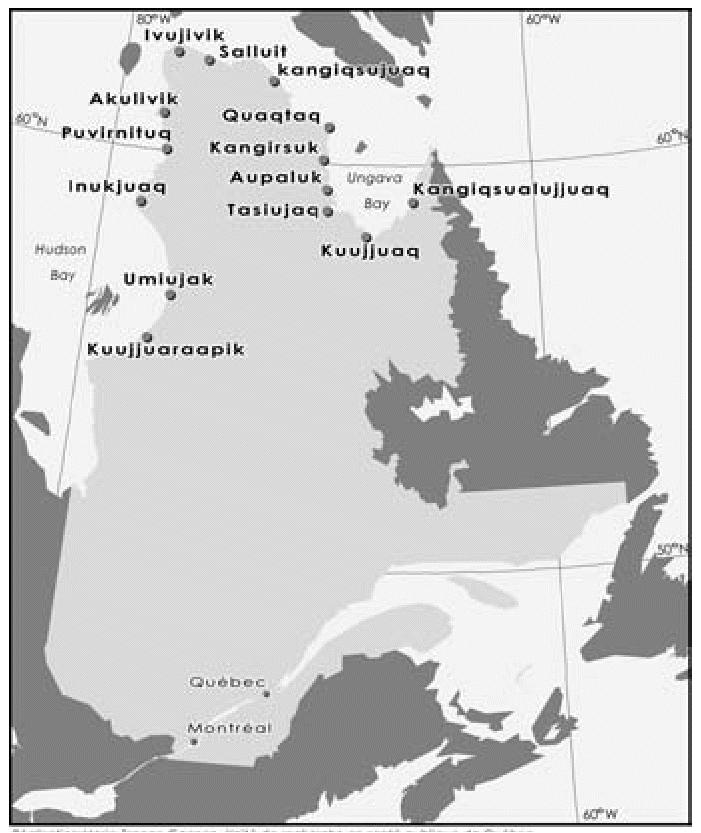
Location of Inuit communities in Nunavik (province of Québec, Canada).

The Nunavik region is located north of the 55th parallel in the province of Québec, Canada, and is composed of 14 isolated villages scattered along the coasts of the Ungava Bay, the Hudson Strait, and the Hudson Bay (Figure 1). The targeted participants for this study were Inuit infants born in Puvirnituq, Inukjuaq, and Kuujjuarapik, the three largest Inuit communities on the Hudson Bay coast in Nunavik. The recruitment procedures have been described elsewhere (Muckle et al. 2001). Briefly, between November 1995 and March 2001, we attempted to contact every pregnant woman after their first prenatal medical visit either by phone or by the community radio (for those without a telephone at home). Pregnant women were invited to meet with our research assistant, and women willing to participate were asked to sign an informed consent form. The study was part of a larger study focusing on environmental contaminants and neurobehavioral development. The study protocol was reviewed and approved by the Nunavik Health and Nutrition Committee and by the ethics committee of Laval University.

### Data collection and biological sampling.

In order to gather biological samples and information on confounding variables, we conducted four interviews: one at midpregnancy (prenatal interview, median of 21 weeks gestation) and three with the infant and the mother at 1, 6, and 11 months postpartum. We collected information on maternal age, breast-feeding duration, socioeconomic status of the care-giver (Hollingshead index), smoking habits during pregnancy, environmental tobacco exposure during the first year of life, number of children living with the participant, village of residence, and day care attendance. Many other characteristics were also documented for the neurobehavioral arm of this cohort but were not included in this study.

We sampled maternal blood at delivery or, when it was impossible, as soon as possible after delivery (median, 2 days postpartum). We also obtained umbilical cord blood at delivery and infant blood at midfollow-up (median, 7.0 months of age). All blood samples were immediately centrifuged and frozen at −80°C. Frozen blood and plasma samples were sent to the Centre de Toxicologie (Institut National de Santé Publique du Québec, Québec City, Canada) every 3–6 months for contaminants and biochemical analyses. Finally, we extensively reviewed the medical charts of the mother and the infant for the pregnancy period and for the infant’s first year of life.

### Determination of OCs.

We determined the concentrations of *p*,*p*′-dichlorodiphenyl-dichloroethylene (DDE) and 14 PCB congeners (International Union of Pure and Applied Chemistry numbers 28, 52, 99, 101, 105, 118, 128, 138, 153, 156, 170, 180, 183, and 187) in plasma samples by high-resolution gas chromatography. OCs were extracted from plasma with ammonium sulfate:ethanol: hexane (1:1:3). The extracts were cleaned on florisil columns, taken to a final volume of 100 μL, and analyzed on an HP-5890 series II gas chromatograph equipped with dual-capillary columns and dual Ni-63 electron-capture detectors (Hewlett-Packard, Palo Alto, CA, USA). We identified peaks by their relative retention times obtained on the two columns. Quality control procedures were described previously (Rhainds et al. 1999). Percent recovery ranged from 89 to 100%, and the detection limit was approximately 0.02 μg/L for all compounds. Coefficients of variation (*n* = 20, different days) ranged from 2.1 to 9.1%. The difference between the concentration of reference material and that found using the analytic method ranged from 10.9 to 3.8%. Because OCs are stored mainly in body fat, all results for contaminants are expressed on a lipid basis.

### Determination of blood lipids.

We measured total cholesterol, free cholesterol, and tri-glycerides in plasma samples by standard enzymatic procedures. Concentrations of phospholipids were determined according to the enzymatic method of Takayama et al. (1977) using a commercial kit (Wako Pure Chemical Industries, Richmond, VA, USA). We estimated the concentrations of total plasma lipids using the formula developed by Phillips et al. (1989).

### Estimation of exposure using plasma concentrations.

In this population, concentrations of maternal OCs are highly correlated with those of cord plasma (*R* = 0.94 for DDE and PCB-153). Because of logistic problems, we were not able to collect cord blood samples for more than half of the participants. Therefore, we used the concentration of OCs in maternal plasma as an estimate of prenatal exposure to OCs. For six subjects, a cord blood sample was available but not a maternal blood sample. For these six subjects, we estimated maternal concentrations from the cord plasma results using linear regression. Postnatal exposure was estimated using plasma concentration of OCs in infant blood at 7 months of age. The concentration of OCs in blood is well correlated with that found in adipose tissues, and it has been shown that either blood or adipose tissue concentrations are valid exposure measurements in epidemiologic studies (Dewailly et al. 1994).

We used PCB-153 concentration (log-transformed) as a surrogate measure for the total PCB burden. PCB-153 is the most abundant PCB congener. Its concentration is strongly correlated with all the moderate-to-heavily chlorinated congeners and with most chlorinated pesticides (except *p*,*p*′-DDT). It has been shown to be a good marker of exposure to most organochlorines in the Arctic (Muckle et al. 2001).

### Medical chart review and incidence of infectious diseases.

Trained research nurses used a standardized questionnaire to review the medical charts of infants for the first 12 months of life. For every diagnosed health problem, we noted the date of diagnosis and the duration of hospitalization (if hospitalized). We also attributed a code corresponding to the *International Classification of Primary Care,* 2nd edition (ICPC-2; World Organization of National Colleges, Academies and Academic Associations of General Practitioners 1998). We then formed four groups of infections: upper respiratory tract infections (URTIs), otitis media, gastrointestinal (GI) infections, and lower respiratory tract infections (LRTIs). We also added a fifth group labeled “all infections,” which included all of the four preceding groups. Because previous studies on OCs and infections in children seem to point toward a greater association between OCs and otitis media compared with other infectious diseases, we excluded ear infections from the URTI category so that otitis and URTIs could be analyzed independently (Chao et al. 1997; Dewailly et al. 2000; Weisglas-Kuperus et al. 2000). The URTI category included streptococcal pharyngitis and tonsillitis, acute upper respiratory tract infection not otherwise specified (NOS), acute rhinitis, head cold, nasopharyngitis, pharyngitis, and coryza. The otitis category included acute suppurative otitis media, otitis media NOS, acute tympanitis, otitis media with effusion, serous otitis media, and glue ear. The LRTI category included acute bronchitis and bronchiolites, acute lower respiratory infection NOS, chest infection NOS, laryngotracheobronchitis, tracheobronchitis, bacterial and viral pneumonia, broncho-pneumonia, influenzal pneumonia, and pneumonitis. The GI infection category included GI infection and dysentery with specified organism, diarrhea or vomiting presumed to be infective, dysentery NOS, and gastric flu.

For every health problem identified, we trusted the diagnosis of the attending physician. When two physicians disagreed, we only recorded the last diagnosis made. In some Inuit communities, nurses are trained to identify and treat benign infections, especially otitis media and URTIs. When the child was not seen by a physician, we recorded the diagnosis of the nurse. We considered two episodes of the same infection type to be separate when there was at least 15 days between the two diagnoses and when it was not specified in the chart that the second episode was related to the first. When an episode of URTI led to a LRTI, we only included the latter in the analysis. We did not attempt to investigate infectious episodes for which treatment at the health center was not sought by the parents. Data on complications or abnormal events during pregnancy, infant sex, and birth weight were also gathered from the medical charts.

### Statistical analyses.

We assigned a value of one-half the detection limit of the analytical method when a compound was not detected in a sample. OC concentrations had log-normal distributions and were log-transformed in all analyses. Therefore, results for contaminants are presented as geometric means. The correlation between contaminant concentrations was evaluated using Pearson’s method on log-transformed values. To evaluate associations between OC exposure and infection incidence rates, we used Poisson regression with quartiles of OC concentration as the main independent variable, and individual incidence rates as the dependent variable (both for bivariate and multivariate analyses). We categorized the exposure using quartiles boundaries, with the first quartile as the group of reference (Table 1). Regression results are, therefore, an estimate of the incidence rate ratios (RRs) for infants in the three highest quartiles of exposure, when infants in each of these quartiles are compared to infants in first quartile. To test the hypothesis of a dose–response association between incidence rates and OC concentrations (*p*-value for trend), we included the contaminant concentration (log-transformed) directly in the model and treated it as a continuous variable.

We based the selection of potential confounding variables on clinical knowledge and a literature review. Every identified potential confounding variable was tested in the model, but only those influencing the incidence rate ratios by > 5% were included in the final model. The variables initially excluded from the model were retested one by one in the final model to ensure that their exclusion did not influence the results. The variables included in the final model were maternal age at delivery (continuous), season of birth, year of birth (category), breast-feeding duration (categories), sex of the infant, socioeconomic status of the caregiver (continuous), smoking during pregnancy (yes/no), number of cigarettes smoked per day during pregnancy (continuous), number of children < 6 years of age living with the infant (continuous), and village of residence. The following variables were excluded from the final model because they did not significantly affect the association of interest: day care frequentation (ever/never), mean hours per week in day care (continuous), maternal omega-3 fatty-acid concentration in blood (continuous), proportion of omega-3 highly unsaturated fatty acids (continuous), number of smokers in the house where the infant resided (continuous), birth weight, gestational age, and reviewer of the medical chart. When postnatal exposure was investigated, we included in the model the infant’s age when the blood sample was drawn. We considered vaccination coverage a potential confounding factor. Information on vaccination was gathered through the review of the medical chart, but information was missing for many children. Preliminary analyses showed that vaccination coverage was not related to contaminant burden. We thus excluded it from the final models.

All modeling results are presented for both the crude model (only exposure categories) and the adjusted model (exposure categories and all the confounding variables mentioned above). Statistical analyses and database management were conducted using the SAS system 8.02 (SAS Institute, Cary, NC, USA). By convention, a *p-*value < 0.05 was considered significant.

## Results

### Recruitment and participation.

During the study period, 417 pregnancies were identified in the targeted communities. Of them, we excluded 47 pregnant women (11.3%) who had already been enrolled in the study during a previous pregnancy and 3 women (0.7%) due to miscarriage, and we were unable to contact 9 women (2.2%). Of the 358 eligible women asked to participate, 110 (30.7%) refused. This refusal rate is comparable with that of other prospective studies with several interviews in populations of low socioeconomic status. Of the 248 women willing to participate, we were unable to review the medical charts of 43 infants for the following reasons: 10 (4.0%) moved to another village, 14 (5.6%) were adopted in another village, 11 (4.4%) because of miscarriage or perinatal mortality, and 8 (3.2%) because the mother withdrew from the study. Finally, we excluded 6 (2.4%) participants because no biological samples were available for exposure analysis. A total of 199 participants were included in the final analyses.

### Population characteristics.

Mothers included in the analysis were mostly from Puvirnituq (45.4%) and Inukjuaq (39.3%). The mean age at delivery was 25.2 years, and most of them smoked during pregnancy (91.4% reported smoking at least 1 cigarette/ day; mean, 10.6 cigarettes/day). Only 2.6% of the infants were not exposed to secondhand smoke during their first year of life. The mean parity was 2.1. There were more males than females (57.6%), and the mean birth weight and length were 3,454 g and 50.3 cm, respectively. Breast-feeding was very common, and only 12.2% were not breast-fed (most of them because they were adopted).

### Incidence of infections.

Incidence proportions and rates for selected infections are shown in Table 2. Otitis media was the most frequent infection diagnosed, with a mean of 2.8 episodes per infant-year, followed by URTIs, with 2.4 episodes per infant-year. During the first year of life, almost all infants had at least one episode of otitis (96.0%), and 17.1% had five episodes or more. LRTIs required hospitalization in 31.4% of cases. More than half of the infants (56.8%) were hospitalized at least once during their first year of life.

### Contaminant burden in plasma.

Table 1 shows the concentration of contaminants in maternal and infant plasma. The geometric mean concentration of the sum of the 14 PCB congeners (∑PCBs) in maternal plasma was 308 μg/kg (range, 60–1,951 μg/kg). The concentration of the ∑PCBs was highly correlated with that of PCB-153 in maternal plasma (*r* = 0.99). The correlation between cord plasma and maternal plasma was also very high, both for the ∑PCBs and for PCB-153 (*r* = 0.95 and 0.94, respectively). The geometric mean concentration for DDE in maternal plasma was 294 μg/kg (range, 54–2,269 μg/kg). The correlation between cord and maternal plasma samples for DDE was also very strong (*r* = 0.94). Mean concentrations of PCBs and DDE were lower in infant plasma compared to those in maternal plasma.

### Prenatal exposure to PCB-153 and infections.

The association between prenatal exposure to PCB-153 and incidence of infections is shown in Table 3. In preliminary analyses we found that the associations between OCs and incidence rates were somewhat stronger during the first 6 months of life. Although this study was designed for a 12-month follow-up, we also present the results for the first 6 months of life. Regarding infections during the first 6 months of life and prenatal exposure to PCBs, we observed statistically significant associations only for LRTIs (3rd quartile; RR = 1.54 and 1.68 for the unadjusted and adjusted models, respectively). Although not statistically significant, almost all other RRs were above the unity. When the four types of infections were combined, the relative rates ranged from 1.19 to 1.20 in the unadjusted model and from 1.17 to 1.27 in the adjusted model. The trend was statistically significant in the adjusted model (*p* = 0.04).

Compared to the first 6 months of life, the effect size was lower when the first 12 months of life were considered, and only GI infections still pointed toward a positive association. The association was significant for the 3rd quartile in the adjusted model only (RR = 1.59). Globally, rate ratios were similar in the unadjusted and adjusted models.

### Prenatal exposure to DDE and infections.

The association between incidence of infections and prenatal exposure to DDE (Table 4) was similar to that observed for exposure to PCB-153. For the first 6 months of life, we detected significant associations with otitis (RR = 1.63, 3rd quartile) and LRTIs (RR = 1.52, 2nd quartile) in the unadjusted model, and with URTIs (RR = 1.56, 2nd quartile) and otitis (RR = 1.83, 3rd quartile) in the adjusted model. The trend was significant for otitis in the unadjusted model (*p* = 0.04) and borderline significant in adjusted model (*p* = 0.07). When the four types of infections were combined, we observed significant associations for the 2nd quartile (RR = 1.49) in the unadjusted model, and for the 2nd (RR = 1.38) and 3rd (RR = 1.33) quartiles in the adjusted model. As observed for PCB exposure, almost all RRs were above the unity.

When considering the first 12 months of life, we observed significant associations for GI infections (RR = 1.49, 2nd quartile) in the unadjusted model, and for URTIs (RR = 1.34, 2nd quartile) and GI infections (RR = 1.59, 2nd quartile) in the adjusted model. For all infections combined, the association reached statistical significance only for the 2nd quartile in the unadjusted model (RR = 1.17).

### Postnatal exposure to OCs and infections.

We used OC concentrations in infant plasma to evaluate the effect of postnatal exposure on incidence of infections (sampling done at a median age of 7.0 months). We observed no association between postnatal exposure and the incidence of infections (data not shown). The only significant association was for PCBs (12-month follow-up, 2nd quartile, RR = 1.19) in the unadjusted model, but the statistical significance was lost when adjustment for confounding was done.

### Effects of exposure to OCs on hospitalization rate.

We found no significant association between prenatal or postnatal exposure and incidence rate of hospitalization for LRTIs (data not shown). However, statistical power was poor because of the limited number of admissions.

## Discussion

Accidental and occupational exposure to PCBs has already been associated with increased susceptibility to infections in infants. Rogan et al. (1988) observed that mothers who were exposed to PCBs through the consumption of contaminated rice oil (Yu-Cheng) reported a higher rate of bronchitis in their children than did control mothers. After examination by two otolaryngologists, the same children were also shown to have a higher prevalence of middle ear diseases than matched controls (Chao et al. 1997). In Japan, Hara (1985) noted that infants born to women who had handled PCBs in a capacitor factory had a higher incidence of colds and GI complaints.

However, evidence of an effect of environmental OC exposure on susceptibility to infection in children is scarce and inconsistent. To our knowledge, the first study addressing this question was conducted in the Great Lakes area (Smith 1984); the author observed that fish consumption during pregnancy (a proxy of PCB exposure) was positively associated with colds, earaches, and flu symptoms in infants. Rogan et al. (1987) followed 900 families in North Carolina (USA) between 1978 and 1982. They reviewed children’s medical charts and did not find any evidence of harmful effects of PCBs or DDE during the first year of life. In the Netherlands, Weisglas-Kuperus et al. (1995) observed no association between PCBs and the number of episodes of rhinitis, bronchitis, tonsillitis, and otitis during the first 18 months of life. However, in the same group of children at 42 months of age, current PCB burden was associated with a higher prevalence of recurrent middle ear infections and chicken pox (Weisglas-Kuperus et al. 2000). Karmaus et al. (2001) also observed a higher risk of otitis media, but the association was only present with the combined exposure to DDE and PCBs. Finally, our laboratory previously reported that exposed Inuit infants had a higher risk of acute otitis media during the first year of life (third tertile of exposure compared to the first) (Dewailly et al. 2000). The association was significant with exposure to DDE and HCB but remained above the unity for PCBs, dieldrin, and mirex.

In this study, we showed that prenatal exposure to some environmental OC contaminants was possibly associated with a higher incidence rate of infections during the first 6 months of life. Although the associations were not always statistically significant because of limited statistical power, infants in the highest quartiles of PCB and DDE exposure had systematically more episodes of infections than their counterparts in the first quartile of exposure. This was mostly observed during the first 6 months of life, as the effect size was lower when infections during the first 12 months of life were considered. Postnatal exposure to OCs was not associated with infection incidence.

In the literature, middle ear infections are the most consistently reported infections associated with prenatal exposure to OCs. In our study, the strongest dose–response relationship was seen with ear infections. However, it is likely that insults of OCs on the developing immune system would result in the increase of incidence of many different types of acute infections and not only ear infections. Consistent with that assumption, our results showed a higher incidence rate for the four most frequent infections in infants in the higher exposure groups, and the rate ratios were similar to that observed for otitis. Furthermore, when these four types of infections were combined, the association was more stable and the magnitude of the dose–response relationship was increased, compared with that of the four types of infection taken separately.

We also observed that the effect of pre-natal exposure was mostly present during the first few months of life and that this effect seemed to vanish after 6 months of life. Furthermore, we found no effect of postnatal exposure to OCs with infections. It has already been suggested that the immune system is vulnerable to immunotoxic compounds during its development and that high maternal burden during pregnancy and lactation could lead to permanent defects on the infant’s immune system (Badesha et al. 1995; Barnett et al. 1987). Our results support the hypothesis of a stronger effect during early infancy, but we were unable to clearly identify any harmful effect persisting after the age of 6 months. After a few months of life, cumulative environmental influences on the immune system may begin to play a larger role, thus increasing the variability of responses to infections. Furthermore, contributions of the OC exposure via breast milk, entangled with the beneficial effect of breast-feeding on infections, might have masked the effect. This could explain in part the discrepancies in results of other studies on OCs and infections because the age of children during disease and exposure assessment varied considerably between studies. Further studies are needed to clarify the time period during which environmental exposure to OCs has a detrimental effect on children health.

In this population, plasma concentrations of many environmentally persistent OCs are strongly correlated (Muckle et al. 2001). Muckle et al. (2001) also showed that concentrations in cord plasma, maternal plasma, and breast milk samples are also strongly correlated. With such exposure, it is therefore not possible to attribute the effect observed to one specific OC compound, nor are we able to unravel the specific contribution of PCB-153 exposure from DDE exposure. Furthermore, our data did not allow us to determine whether the association between DDE and infections was due to an immune modulation property of DDE, to co-linearity with PCB-153, or both.

We used a review of the medical charts to evaluate disease frequency. There is only one health center in each of the three Inuit communities included in this study, and participants almost always go to that heath center when they seek medical attention; copies of consultations performed elsewhere are routinely requested to complete medical charts. We are therefore confident that we have reviewed a majority of the medical consultations sought by the participants. Nevertheless, we did not attempt to verify every diagnosis, nor did we try to inquire about infections for which medical attention was not sought by the parents. Furthermore, we did not find a suitable proxy for the propensity to go to the clinic when symptoms were present (health services are free of charge in Canada). Our results are therefore likely to be an underestimation of the true incidence. This underestimation is expected to be present for benign infection, but is unlikely to be significant for LRTIs. This underestimation may be associated with traditional lifestyle, and thus with OC exposure, but the direction of the bias is unknown. However, if such a bias was present, we could assume that it would have persisted beyond 6 months of age. RRs for the 12-month follow-up are close to unity; therefore, the bias seems to have little effect on our results.

Because of the relatively small number of subjects involved (*n* = 199), our results must be regarded with caution. Many factors can greatly influence the rate of acute infections. We have assessed several potential confounding factors, but unknown factors might still be present. Specifically, we cannot rule out the possibility that the infants in the lowest exposure group (first quartile) had better general health due to an unknown cause or simply due to chance. This would have resulted in RRs above the unity for the three highest quartiles of exposure without any dose–response association, which is similar to what we observed. This should be kept in mind in interpreting our results.

The high rate of infectious episodes in young Inuit children has been observed in northern Canada, the United States (Alaska), and Greenland (Banerji et al. 2001; Holman et al. 2001; Koch et al. 2002; Proulx 1988; Wainwright 1996). Many cultural, environmental, and economical factors contribute to this situation. Our study population is no exception, with a mean of almost nine infection-related medical consultations per infant during the first 12 months of life. In the context of such a high rate of infections, rate ratios of around 1.25, like the ones observed in this study, could have a tremendous impact on the public health of this population. This is the second study identifying a possible association between acute infections and prenatal exposure to OCs in Nunavik. However, the relatively small number of subjects raises the possibility of an association that could be due to chance. To further clarify the potential contribution of persisting contaminants in the high infection rate of these children, we are currently conducting another study in which a third cohort of Inuit children from the same population is being followed during the first 5 years of life. Other studies are also needed to identify which immune mechanisms are involved and to better understand the role of maternal passive immunity in these infants. In the meantime, awareness and precautions regarding the selection of marine food items before and during pregnancies are warranted.

## Figures and Tables

**Table 1:** Contaminant concentrations in plasma (μg/kg lipid-based).

				Quartile boundaries			
Contaminant	Percent detected	Geometric mean (95% CI)	Range	1st	2nd	3rd	4th
Maternal plasma (*n* = 199)							
∑ PCBs	NA	308 (279–340)	59.6–1,951	< 190	190–296	296–500	> 500
PCB-153	100	102 (91.4–113)	14.6–709	< 57.6	57.6–98.4	98.4–170	> 170
DDE	100	294 (267–324)	54.3–2,269	< 183	183–281	281–472	> 472
Infant plasma (*n* = 172)							
∑PCBs	NA	259 (218–307)	26.9–3,801	< 99.0	99.0 –283	283–609	> 609
PCB-153	96.5	76.1 (62.4–92.9)	ND–1,441	< 28.0	28.0–95.3	95.3–199	> 199
DDE	100	256 ( 214–307)	15.6–4,386	< 100	100–355	355–618	> 618

Abbreviations: CI, confidence interval; NA, not applicable; ND, not detected.

**Table 2:** Incidence proportion and mean infection incidence rate for all participants (*n* = 199).

			Percentage of participants who had at least		
Infection	Mean incidence (episodes per person × year)	Percentage of episodes requiring hospitalization	1 episode	3 episodes	5 episodes
URTIs	2.4 ± 1.7	1.3	90.0	42.7	12.6
Otitis media	2.8 ± 1.7	0	96.0	52.8	17.1
GI infections	1.0 ± 1.1	3.4	58.8	10.6	0.5
LRTIs	1.7 ± 1.7	31.4	73.4	26.6	5.5

**Table 3:** Incidence RR of each PCB-153 quartile of prenatal exposure compared to the first quartile.

	Unadjusted (*n* = 199)				Adjusted (*n* = 177)*^a^*			
	Incidence RR (95% CI)*^b^*				Incidence RR (95% CI)*^b^*			
Infection type	2nd quartile (*n* = 50)	3rd quartile (*n* = 50)	4th quartile (*n* = 50)	*p*-Value for trend*^c^*	2nd quartile (*n* = 40)	3rd quartile (*n* = 46)	4th quartile (*n* = 45)	*p*-Value for trend*^c^*
6-Month follow-up								
URTIs	1.08 (0.76–1.55)	0.98 (0.68–1.41)	1.19 (0.84–1.68)	0.69	1.08 (0.69–1.67)	1.08 (0.71–1.65)	1.32 (0.87–2.00)	0.22
Otitis media	1.33 (0.85–2.07)	1.15 (0.73–1.82)	1.30 (0.83–2.02)	0.17	1.11 (0.65–1.89)	0.99 (0.59–1.66)	1.39 (0.82–2.35)	0.17
GI infections	1.63 (0.80–3.34)	1.31 (0.62–2.76)	1.55 (0.75–3.20)	0.33	1.89 (0.78–4.56)	1.52 (0.65–3.54)	1.54 (0.66–3.60)	0.38
LRTIs	1.12 (0.71–1.76)	1.54 (1.01–2.35)*	1.01 (0.63–1.61)	0.61	1.16 (0.65–2.09)	1.68 (1.00–2.81)*	1.18 (0.68–2.04)	0.38
All infections*^d^*	1.19 (0.95–1.50)	1.18 (0.94–1.48)	1.19 (0.95–1.50)	0.14	1.17 (0.88–1.55)	1.19 (0.92–1.54)	1.27 (0.98–1.66)	0.04*
12-Month follow-up								
URTIs	0.93 (0.72–1.20)	0.87 (0.67–1.13)	1.12 (0.88–1.43)	0.81	0.99 (0.71–1.36)	0.96 (0.71–1.29)	1.23 (0.92–1.65)	0.29
Otitis media	1.05 (0.83–1.32)	0.97 (0.76–1.22)	0.94 (0.75–1.20)	0.89	1.02 (0.77–1.35)	0.89 (0.68–1.17)	0.97 (0.73–1.28)	0.89
GI infections	1.27 (0.86–1.88)	1.22 (0.82–1.82)	1.05 (0.69–1.58)	0.81	1.53 (0.94–2.49)	1.59 (1.01–2.49)*	1.26 (0.78–2.04)	0.29
LRTIs	0.88 (0.65–1.19)	1.08 (0.81–1.45)	0.96 (0.71–1.29)	0.48	0.86 (0.57–1.28)	1.10 (0.78–1.55)	1.03 (0.72–1.48)	0.36
All infections*^d^*	1.00 (0.87–1.15)	0.99 (0.86–1.14)	1.01 (0.88–1.16)	0.67	1.02 (0.86–1.21)	1.01 (0.86–1.19)	1.08 (0.92–1.28)	0.24

CI, confidence interval.

a Model included mother’s age, season of birth, year of birth, breast-feeding duration, sex, socioeconomic status of the caregiver, tobacco use during pregnancy, village of residence, and number of children living with the participant.

b Incidence RR when the given quartile was compared to the first quartile of exposure (Poisson regression).

c *p*-Values for trends were calculated by Poisson regression in which the contaminant concentration (lipid-based) was entered as a continuous variable (log-transformed).

d Only infections with a mean incidence > 1.0 episode/year/infant were included; see details in “Materials and Methods”).

* *p* < 0.05.

**Table 4:** Incidence RR of each DDE quartile of prenatal exposure compared to the first quartile

	Unadjusted (*n* = 199)				Adjusted (*n* = 177)*^a^*			
	Incidence RR (95% CI)*^b^*				Incidence RR (95% CI)*^b^*			
Infection type	2nd quartile (*n* = 50)	3rd quartile (*n* = 50)	4th quartile (*n* = 50)	*p*-Value for trend*^c^*	2nd quartile (*n* = 40)	3rd quartile (*n* = 46)	4th quartile (*n* = 45)	*p*-Value for trend*^c^*
6-Month follow-up								
URTIs	1.50 (1.05–2.13)	1.06 (0.72–1.55)	1.19 (0.82–1.73)	0.91	1.56 (1.05–2.33)*	1.15 (0.75–1.75)	1.40 (0.90–2.16)	0.24
Otitis media	1.27 (0.79–2.05)	1.63 (1.04–2.57)*	1.50 (0.95–2.38)	0.04*	1.03 (0.59–1.77)	1.83 (1.09–3.07)*	1.55 (0.90–2.68)	0.07
GI infections	2.16 (1.02–4.55)*	1.76 (0.81–3.82)	1.67 (0.76–3.64)	0.34	1.91 (0.84–4.35)	1.66 (0.69–3.97)	1.35 (0.54–3.42)	0.58
LRTIs	1.52 (1.00–2.32)*	1.01 (0.64–1.59)	1.01 (0.64–1.59)	0.75	1.40 (0.86–2.29)	1.22 (0.72–2.05)	0.96 (0.55–1.66)	0.89
All infections*^d^*	1.49 (1.19–1.87)*	1.23 (0.97–1.55)	1.25 (0.99–1.57)	0.22	1.38 (1.07–1.78)*	1.33 (1.03–1.73)*	1.27 (0.96–1.67)	0.11
12-Month follow-up								
URTIs	1.27 (0.98– 1.63)	1.03 (0.79– 1.34)	1.11 (0.85–1.44)	0.85	1.34 (1.00–1.78)*	1.09 (0.81–1.47)	1.30 (0.96–1.78)	0.27
Otitis media	1.00 (0.79–1.27)	1.12 (0.89–1.42)	1.08 (0.85–1.36)	0.36	0.89 (0.68–1.17)	1.08 (0.83–1.41)	1.02 (0.76–1.35)	0.72
GI infections	1.49 (1.00–2.23)*	1.30 (0.86–1.96)	1.20 (0.79–1.82)	0.98	1.59 (1.03–2.47)*	1.27 (0.81–2.00)	1.43 (0.87–2.34)	0.59
LRTIs	1.15 (0.85–1.55)	0.96 (0.70–1.30)	1.05 (0.78–1.42)	0.89	1.07 (0.75–1.51)	0.98 (0.69–1.40)	1.00 (0.69–1.45)	0.99
All infections*^d^*	1.17 (1.02–1.35)*	1.08 (0.93–1.24)	1.09 (0.95–1.26)	0.59	1.13 (0.97–1.33)	1.08 (0.92–1.26)	1.13 (0.95–1.34)	0.38

CI, confidence interval.

a Model included mother’s age, season of birth, year of birth, breast-feeding duration, sex, socioeconomic status of the caregiver, tobacco use during pregnancy, village of residence, and number of children living with the participant.

b Incidence RR when the given quartile was compared to the first quartile of exposure (Poisson regression).

c *p*-Values for trends were calculated by Poisson regression in which the contaminant concentration (lipid-based) was entered as a continuous variable (log-transformed).

d Only infections with a mean incidence > 1.0 episode/year/infant were included; see details in “Materials and Methods”).

* *p* < 0.05.
